# Supplementary material for: Electrically tunable collective motion of dissipative solitons in chiral nematic films
Source: Nat Commun. 2022 Apr 19;13:2122. doi: 10.1038/s41467-022-29831-2 (PMC9018705; doi:10.1038/s41467-022-29831-2)
Supplement: Supplementary file 1 — Supplementary Information [file 41467_2022_29831_MOESM1_ESM.pdf]

Electrically tunable collective motion of dissipative solitons in chiral nematic films

[1]Yuan Shen and [1]Ingo Dierking*
[1]Department of Physics and Astronomy, School of Natural Sciences, University of Manchester, Oxford Road, Manchester, M13 9PL, United Kingdom
E-mail: ingo.dierking@manchester.ac.uk

Supplementary Figures

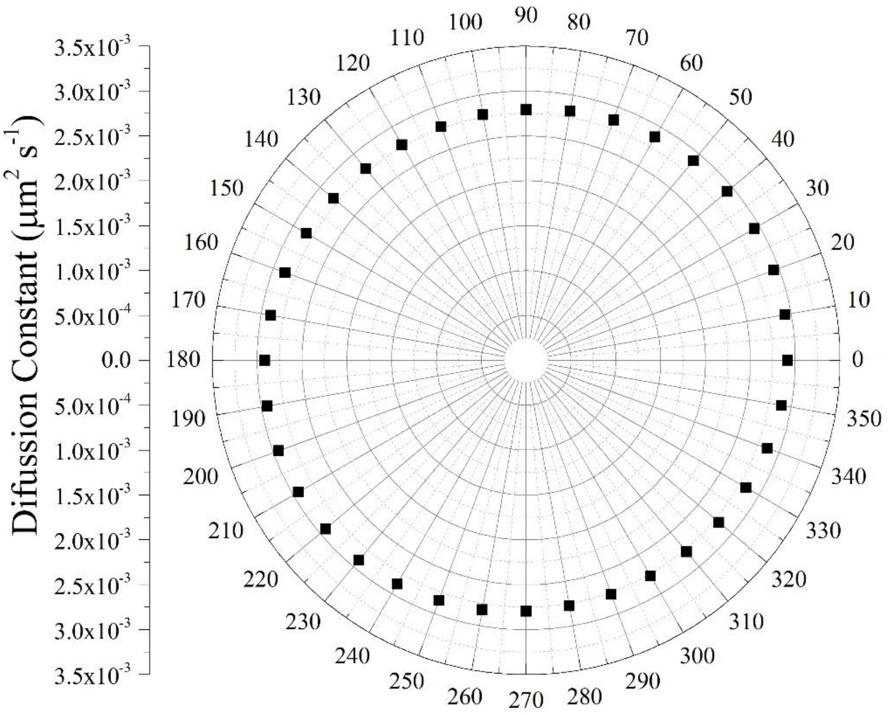

Fig. S1 Angular dependencies of the diffusion coefficient of a colloidal micro-particle in a chiral nematic. The colloidal micro-particle (diameter ~3 μm) is dispersed in a chiral nematic (*p* ~ 2 μm) which is filled in a cell (*d* ~ 10 μm) with planar alignment. The sample is heated to 75 °C.

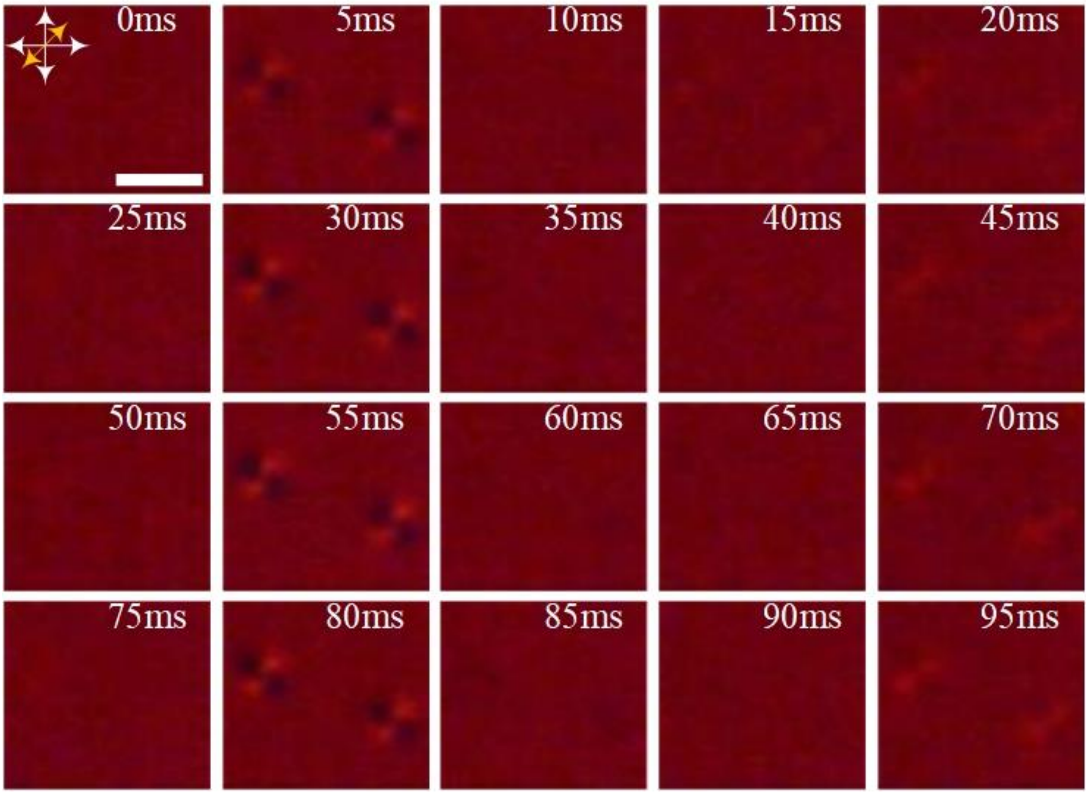

Fig. S2 Time series of polarizing micrographs of directrons. The directrons are modulated by an AC electric field of U = 20 V, f = 20 Hz. The corssed white arrows represent polarizers and the yellow arrow represent the optical axis of the λ-plate. Scale bar 10 μm.

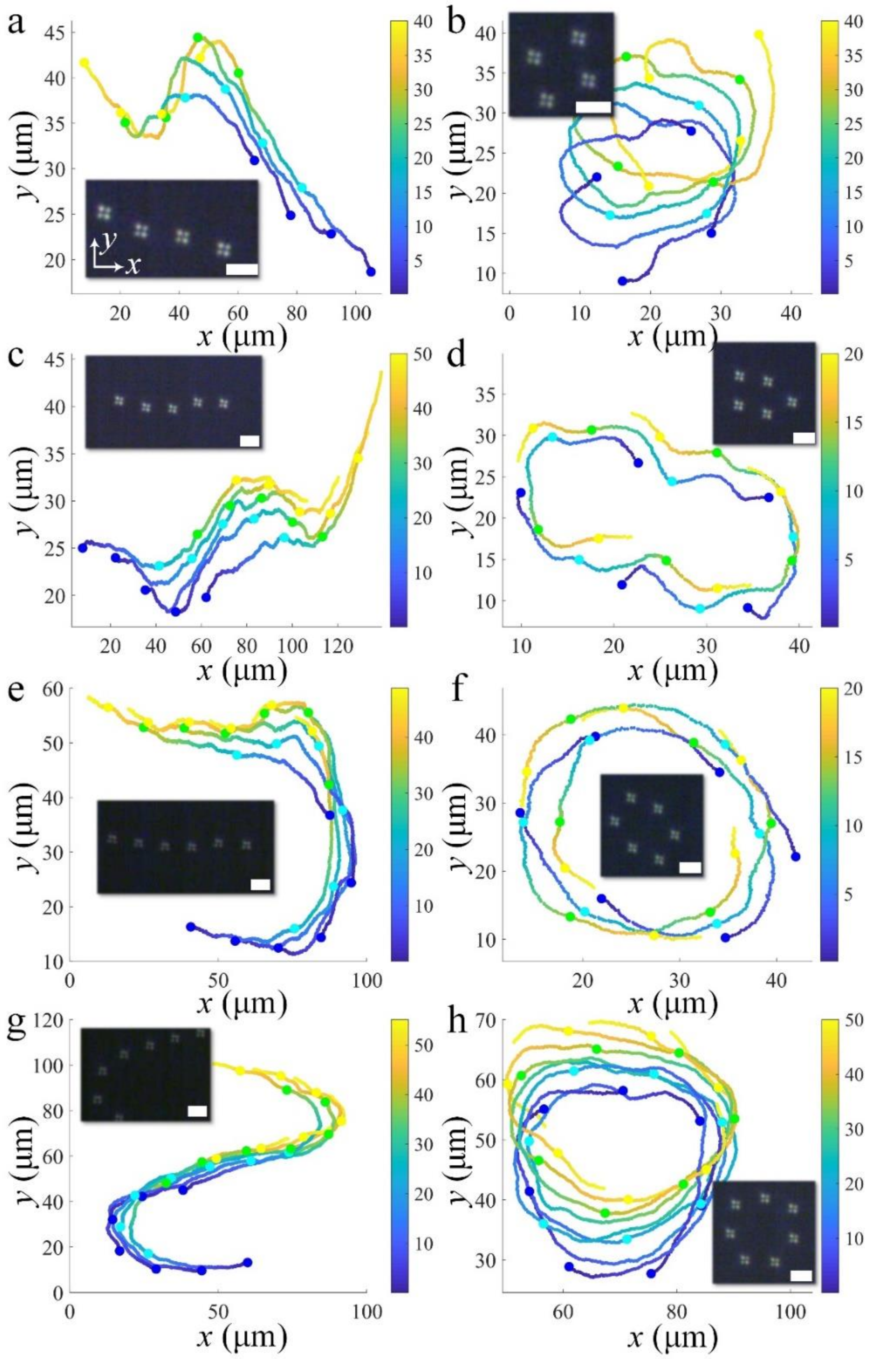

Fig. S3 Dynamics of directron flocks. Trajectories of different number of directrons colored with time corresponding to the color bars (unit (s)) at $U = 15.4$ V, $f = 100$ Hz. **a** and **b**, four directrons; **c** and **d**, five directrons; **e** and **f**, six directrons; **g** and **h**, seven directrons. In **a**, **c**, **e**, and **g**, the directrons form linear chains which move coherently. In **b**, **d**, **f**, and **h**, the directrons form closed loops which rotate continuously about their rotation axes. The insets show the micrographs of the corresponding directrons. Scale bar 10 μm. Polarizers are parallel to the *x*- and *y*-axes, respectively.

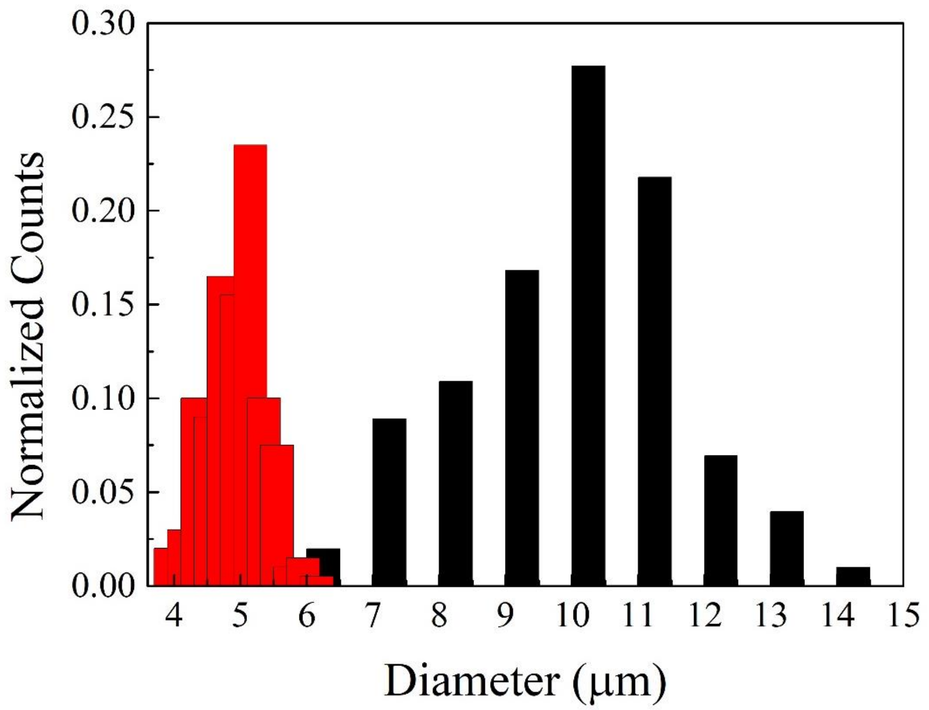

Fig. S4 Directron size distribution. The red bars represent the directrons in planar alignment cells with cell gap d ~ 9.5 μm, U = 20 V, f = 100 Hz. The black bars represent the directrons in planar alignment cells with cell gap d ~ 19.7 μm, U = 100 V, f = 500 Hz.

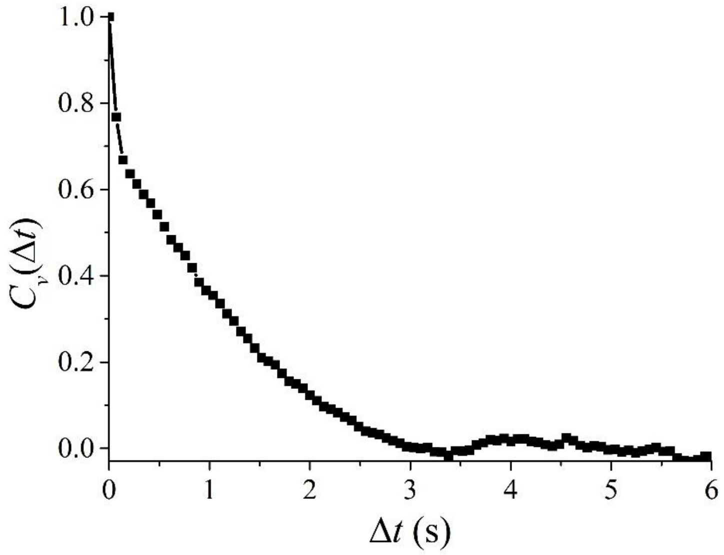

Fig. S5 Temporal velocity correlation function. The temporal velocity correlation function of directrons corresponding to Fig. 9. U = 100 V, f = 500 Hz.

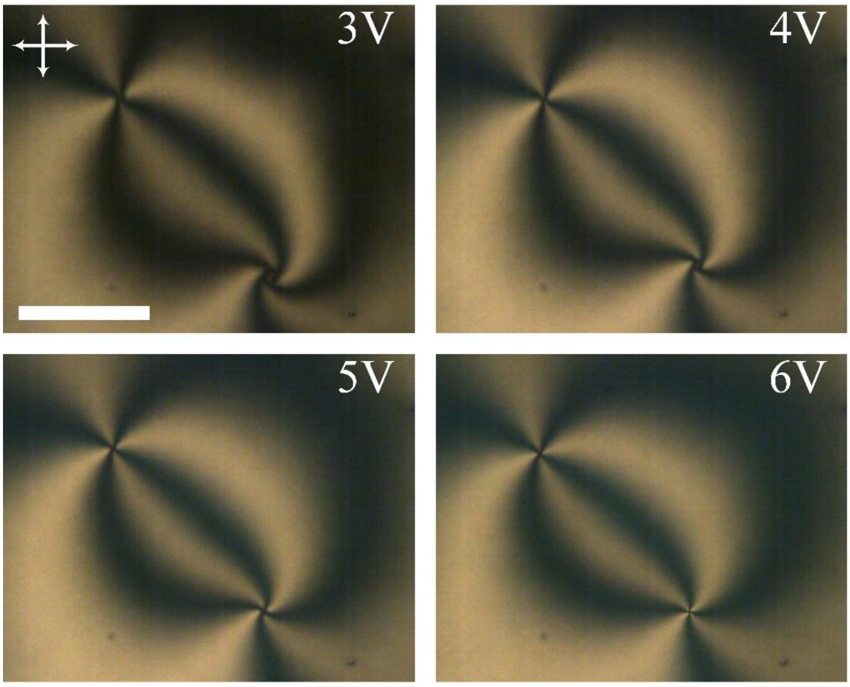

Fig. S6 POM image of a pair of umbilic defects at varied voltages. Demonstration of the size variation of defect cores with increasing voltage. $f = 100$ Hz. Scale bar 100 μm. The crossed white arrows represent the polarizers.

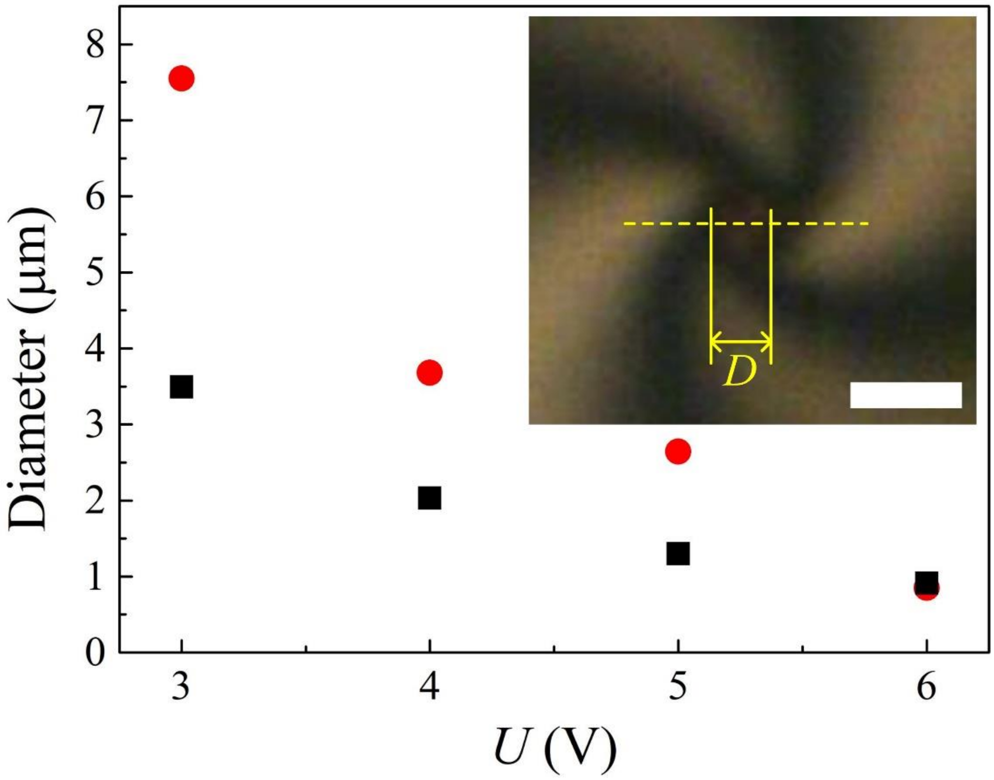

Fig. S7 The voltage dependence of the diameter (*D*) of the umbilic defect core. The red circles represent the $s = +1$ defects and the back squares represent the $s = -1$ defects. The inset shows the POM images of an umbilic defect. The diameter is measured as the width of the cross-section of the defect core as indicated by the yellow lines. Scale bar 10 μm.

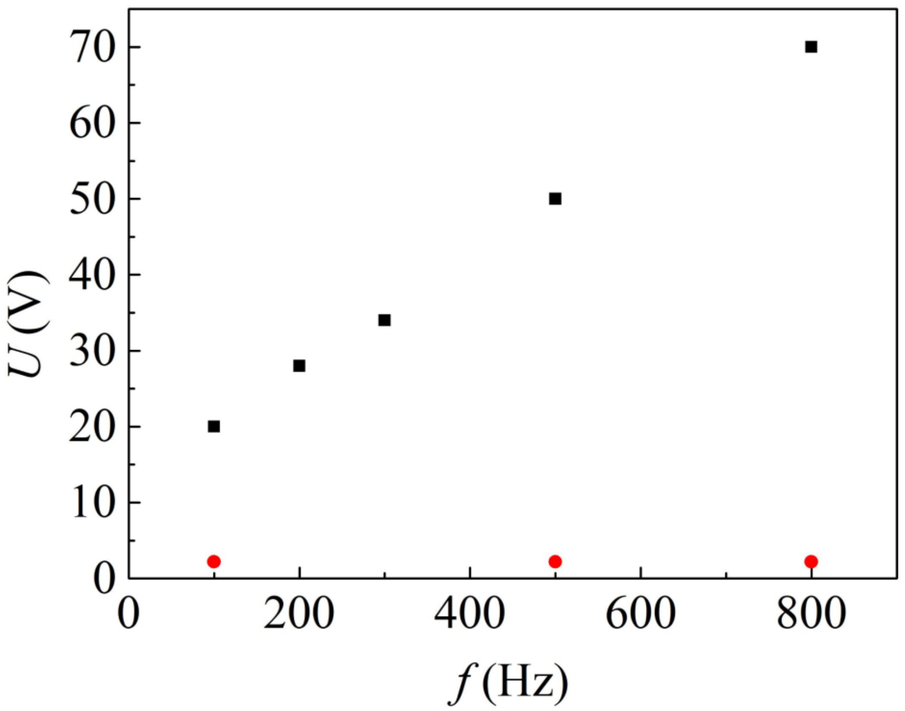

Fig. S8 Frequency dependence of voltage thresholds of directrons. Voltage thresholds of directrons (black squares) and Freedericksz transition (red circles) in cells of homeotropic alignment (p ~ 10 μm, d ~ 9.4 μm).
