# Supplementary material for: Electrically tunable collective motion of dissipative solitons in chiral nematic films
Source: Nat Commun. 2022 Apr 19;13:2122. doi: 10.1038/s41467-022-29831-2 (PMC9018705; doi:10.1038/s41467-022-29831-2)
Supplement: Supplementary file 3 — Description of additional Supplementary File [file 41467_2022_29831_MOESM3_ESM.pdf]

Descriptions of additional supplementary Data files

Supplementary Movie 1. Collision of two directrons at U = 15.4 V, f = 100 Hz. The movie is played at 2× the original speed.

Supplementary Movie 2. Dynamics of flocks of directrons composed of different number of directrons at U = 15.4 V, f = 100 Hz. The movie is played at 10× the original speed.

Supplementary Movie 3. Formation of flocks of directrons at U = 16.2 V, f = 100 Hz. The movie is played at 5× the original speed.

Supplementary Movie 4. Fusion and fission of directron flocks at U = 15.4 V, f = 100 Hz. The movie is played at 5× the original speed.

Supplementary Movie 5. Emergence of large-scale coherent directional motion of directrons at U = 20 V, f =100 Hz. The movie is played at 10× the original speed.

Supplementary Movie 6. Collective motions of directrons at U = 20 V, U = 30 V and U = 40 V, respectively. f = 100 Hz.

Supplementary Movie 7. “Turbulent” swimming pattern of directrons at U = 100 V, f = 500 Hz.

Supplementary Movie 8. Circulating motion of directrons around a s = +1 defect at U = 30 V, f = 100 Hz.

Supplementary Movie 9. Circulating motion of directrons around a micro- particle at U = 40 V, f = 60 Hz.
